# Pushing the Frontiers: Optogenetics for Illuminating the Neural Pathophysiology of Bipolar Disorder

**DOI:** 10.7150/ijbs.84923

**Published:** 2023-08-28

**Authors:** Lingzhuo Kong, Xiaonan Guo, Yuting Shen, Le Xu, Huimin Huang, Jing Lu, Shaohua Hu

**Affiliations:** 1 Department of Psychiatry, the First Affiliated Hospital, Zhejiang University School of Medicine, Hangzhou 310003, China.; 2 School of Psychiatry, Wenzhou Medical University, Wenzhou 325000, China.; 3 The Key Laboratory of Mental Disorder's Management in Zhejiang Province, Hangzhou 310003, China.; 4 Brain Research Institute of Zhejiang University, Hangzhou 310003, China.; 5 Zhejiang Engineering Center for Mathematical Mental Health, Hangzhou 310003, China.; 6 Department of Neurobiology, NHC and CAMS Key Laboratory of Medical Neurobiology, School of Brain Science and Brian Medicine, and MOE Frontier Science Center for Brain Science and Brain-machine Integration, Zhejiang University School of Medicine, Hangzhou 310003, China.

**Keywords:** Bipolar disorder, optogenetics, pathophysiology, frontal-limbic system, biological rhythms, animal model

## Abstract

Bipolar disorder (BD), a disabling mental disorder, is featured by the oscillation between episodes of depression and mania, along with disturbance in the biological rhythms. It is on an urgent demand to identify the intricate mechanisms of BD pathophysiology. Based on the continuous progression of neural science techniques, the dysfunction of circuits in the central nervous system was currently thought to be tightly associated with BD development. Yet, challenge exists since it depends on techniques that can manipulate spatiotemporal dynamics of neuron activity. Notably, the emergence of optogenetics has empowered researchers with precise timing and local manipulation, providing a possible approach for deciphering the pathological underpinnings of mental disorders. Although the application of optogenetics in BD research remains preliminary due to the scarcity of valid animal models, this technique will advance the psychiatric research at neural circuit level. In this review, we summarized the crucial aberrant brain activity and function pertaining to emotion and rhythm abnormities, thereby elucidating the underlying neural substrates of BD, and highlighted the importance of optogenetics in the pursuit of BD research.

## Background

Bipolar disorder (BD), a crippling mental disorder characterized by mood cycling of manic (e.g., impulsivity, reduced need for sleep, increased energy and hyperactivity, reduced anxiety and depression) and depressive (e.g., helplessness, anhedonia, and reduced energy and activity) episodes, affects 1-4% of the population worldwide 1-3. The risk of suicide in BD patients in terms of emotional extremes was 20-30 times higher compared with the non-BD population 4, 5, creating a giant burden for social healthcare and medical institutions 6.

It is imperative to understand the neural mechanisms of BD and identify potential targets for therapeutic interventions. Therefore, animal models developed by drug, gene knockout, and chronic stress are applied as feasible research methods. Although BD patients usually show key features in emotional fluctuation and disrupted biological rhythms, the animal models to date cannot easily mimic the switching emotional phenotypes with high validity, which hinders the understanding of BD mechanism 7. With the progression of optogenetics, exploring the possibilities of optogenetic control to promote research on BD mechanism represents a promising frontier.

Optogenetics, a combination of genetic and optical methods, has been utilized to regulate the activity of living cells in the real-time through ion flux under optical control 8, 9. Channelrhodopsins [ChR], archaerhodopsin [Arch], and halorhodopsins [HR] are the most frequently employed opsins to modulate the neural activity 10-12. After virally expressing the mentioned light-sensitive protein in certain brain regions through stereotactic injection, it allows the certain ion flux passing through the membrane when the specific light wavelength is given. This ion flux thus drives the alteration of neural excitability by changing the membrane potential. Under this circumstance, light, acting as the effector, achieves the manipulation of the activity in the desired neural ensembles both *in vitro* and *in vivo* with an experiment-tailored light pattern. Therefore, optogenetics shows advantages to operate with high spatial and temporal resolution 13, 14. To date, this precise temporal and spatial light-control system has been integrated with other complimentary methods, such as electrophysiology, to further elucidate the psychiatric pathology 8.

Since dysfunction of the specific regions in the brain has been tremendously implicated in BD pathophysiology, it is necessary to mimic and rescue the dynamic emotional symptoms (namely, the emotion-related behaviors) of modelled animals that are consistent with clinical manifestations of BD. Therefore, in this review, we summarized the potential targets for optogenetic intervention from human evidence to back up the BD animal researches, and tried to shed light on the application of optogenetics in animal models on elucidating the possible mechanisms of BD.

## Neural basis of BD pathophysiology

As the brain is pivotal in mediating emotion and guiding behavior, it is supposed that some unique alterations in related brain regions sculpt the core symptoms in BD patients such as consistent emotional instability and disrupted biological rhythms. Growing evidence has demonstrated that the pathophysiology of BD probably lies in the structural and functional changes of frontal-limbic system (including the hippocampus, amygdala, septum, orbitofrontal gyrus, hypothalamus, dentate gyrus, and cingulate gyrus) 15, as well as the suprachiasmatic nucleus (SCN) (SCN belongs to the limbic system, but we separately discuss this peculiar nucleus in view of its specific rhythm-pacemaker role) 16. The former frontal-limbic system is highly associated with affective regulation. It has also been demonstrated that dysregulated brain activity and the neurotransmitter pathways in this system might directly delineate morbid emotions in BD patients. The latter SCN, acting as a circadian rhythm center, is manipulated by regulatory genes including* CLOCK*. Depending on this circadian gene expression, it in turn mediates the metabolic balance, which may also correlate with the emotional abnormalities in BD 16, 17. In this review, we mainly focused on the frontal-limbic system and the SCN.

## Abnormalities in the frontal-limbic system

### Alterations in the brain activity

The first pathophysiological model of mood disorder in humans originated from the “limbic-cortical-striatal-pallidal-thalamic tract” model 18, which has now evolved into the “frontal-limbic” model. Anatomically, extensive dense reciprocal connections have been confirmed in the frontal-limbic system to predominantly participate in emotion mediation with top-down and bottom-up mechanisms 19, 20. Morbidity of this system has been examined in the affective disorders, including BD. It seems that alterations in the brain activity (manifesting as changes in the functional connectivity via neuroimaging) within the frontal-limbic system, especially the compromised integrity in the frontal-subcortical and prefrontal-limbic areas, are overwhelmingly involved in the pathogenesis of BD 21-30. Herein, we summarize the crucial alterations in the brain activity in the frontal-limbic system of diagnosed BD patients.

The frontal lobe is well known for its necessity in modifying the moods, and impairment of the frontal lobe negatively affects emotional cognition 31. Studies have illustrated that the lingual gyrus, insula, putamen, bilateral superior frontal gyrus, and superior frontal gyrus have significantly altered low-frequency fluctuations in BD subjects compared with those in healthy controls 32, 33. Notably, the hyperactivity of ventral cingulate cortex is more strongly associated with emotion state rather than medication results. Given the mood instability of BD, an earlier meta-analysis showed that the activation of the prefrontal cortex was attenuated in the manic state of BD, while the activation of the limbic system remained consistently enhanced across all the emotional states 34. Specifically, decreased engagement of the medial orbitofrontal frontal cortex also plays a role during explicit regulation of the negative emotions of BD, leading to hopelessness that contributes to the risk of suicide 35. Nevertheless, challenge remains since the frontal lobe is composed of heterogeneous functional components. Therefore, the role of specific subregions is still vague, necessitating more precise voxel-based analysis or invasive research using optogenetics in animal models.

The amygdala, which is located in the limbic system, has long been illustrated in the emotional morbidity of BD. Evidence has shown that some regions in the prefrontal cortex, namely, the ventrolateral prefrontal cortex (VLPFC), the dorsolateral prefrontal cortex (DLPFC), and the anterior cingulate cortex (ACC), cooperate with the amygdala in the limbic system in processing emotion 36-38. Therefore, the amygdala is able to extract emotional stimulus information from upstream brain regions and environmental stimuli, thus playing a central role in emotion processing and initiating arousal reactions to incoming information immediately. To achieve emotion regulation, the activation of the amygdala stimulates the fast-acting autonomic nervous system and the slow-acting hypothalamic-pituitary-adrenal axis through neuronal projections to the brainstem and hypothalamus, allowing adaptive modulation of stress responses 39. Pathologically, different levels of amygdala activation can suitably reflect different mood states of BD 40, 41. Specifically, heightened activation of the amygdala was illustrated in the manic state of BD, especially in the left amygdala while the patients were watching the fearful faces 41, indicating that the amygdala might regulate extreme emotions such as fear. It has also been reported that hyperactivation of the right amygdala while viewing fearful faces is correlated with a higher risk of developing BD 42-44. A trend of left amygdala hyperactivation was observed in the depressive state of BD 45, but without statistical significance. In addition, the activity of the PFC and amygdala was detected with less resilience in euthymic BD subjects than in healthy controls 46-49.

**Figure 1:**
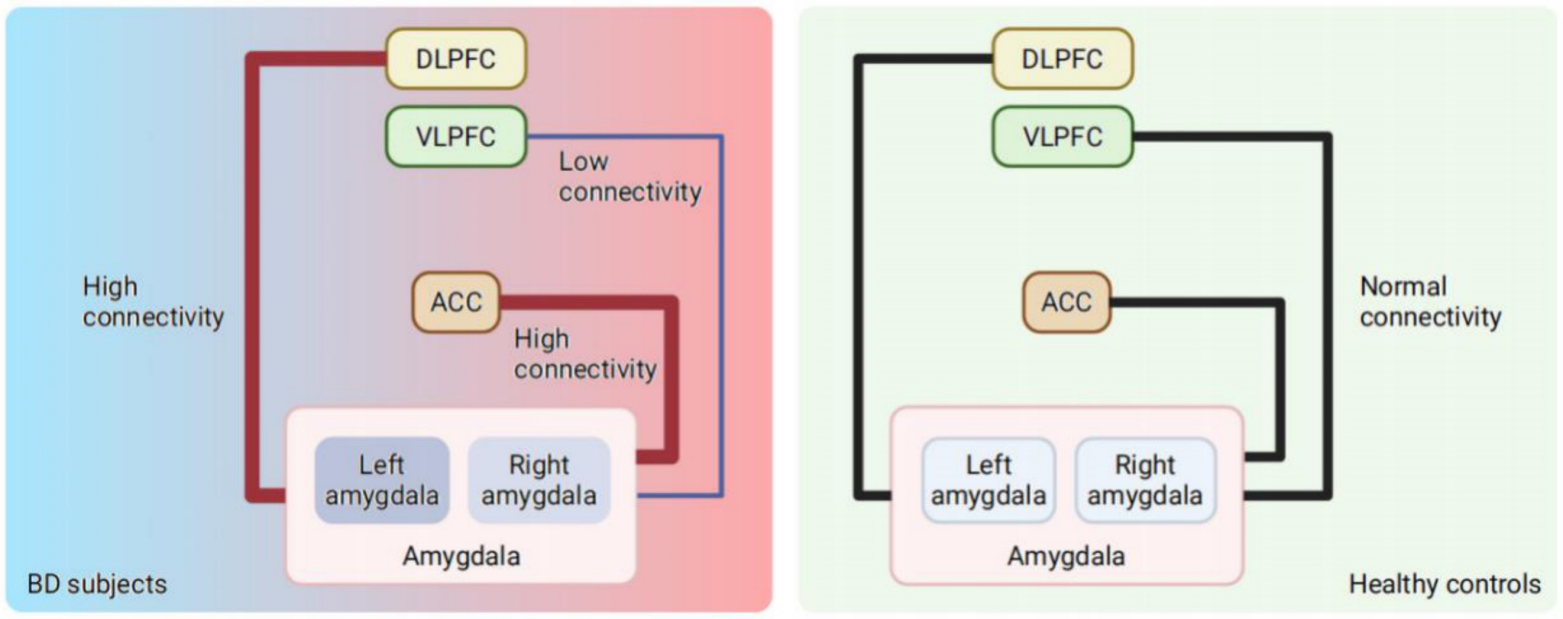
Changes in the amygdala activity and connectivity in BD subjects compared with healthy controls*. *Both increased and decreased amygdala activation have been found in euthymic BD and healthy controls, which is not showed in this figure. Evidence showed increased activation of amygdala in BD subjects compared to healthy controls. The activity of left amygdala increased significantly during mania stage of BD, while the trend of increased activity of left amygdala has been observed in BD depression. Higher connectivity between the amygdala and the DLPFC, as well as lower connectivity between the amygdala and VLPFC was found in BD, and heightened connectivity was also detected between the amygdala and ACC. BD = bipolar disorder; VLPFC = ventrolateral prefrontal cortex; DLPFC = dorsolateral prefrontal cortex; ACC = anterior cingulate cortex.

With regard to functional connectivity, BD patients exhibit higher connectivity of the amygdala with the ACC and DLPFC 50, as well as decreased connectivity between the VLPFC and amygdala 30, 51, 52, (**Figure 1**) which is possibly attributed to developmental failure 30 or neuroinflammation 53. The ACC and DLPFC can regulate the negative emotional responses from the amygdala to resolve conflicts. At the same time, the activity of the ACC is directly attentionally modulated by the DLPFC. In fact, the left ACC and right hippocampus in BD patients showed decreased resting-state functional connectivity between the left ACC and the left orbitofrontal cortex compared with those of healthy controls 24. For clinical practice, such connectivity changes revealed in subcortical, anterior temporal and ventral prefrontal regions responding to emotional events in BD patients have been considered biomarkers for the vulnerability and expression of BD 29.

Note that numerous studies have disclosed that the activity of brain regions in the frontal lobe and limbic system as well as the interplay between them are together presumed to contribute to the irregular emotion of BD patients. However, the causality between aberrant frontal-limbic systems and the pathology of BD remains elusive. All these discoveries in humans provide valuable target brain regions or circuits to promote the further dissection direction of neural mechanisms in animals.

### Dysregulated dopaminergic within the frontal-limbic system

In parallel with altered activity and functional connectivity, dysregulated neurotransmitters have also been disentangled in BD. As the dysregulation of reward processing has been demonstrated in BD patients 60, dopamine, which has been demonstrated to be crucially involved in transforming emotional value or coding appetite, attracts the most attention 54.

Several functional magnetic resonance imaging studies employing reward tasks indicated the morbid neural activity in the frontal-limbic system of BD patients. In detail, during the hypomanic/manic period, the frontal-striatal neural circuit, which is highly involved in reward processing and approach-related effect, was elevated activated 55-58. The metabolism rate decreased in the prefrontal cortex of bipolar mania patients, but increased in other frontal-limbic regions including dorsal cingulate cortex, striatal regions, nucleus accumbens (NAc), etc 59.

Beyond functional neuroimaging, molecular evidence was also gathered. In clinical practice, D2 blocker antipsychotics treatment is effective in many BD patients, which hints the potential pathology-involved targets. Experiments conducted with positron emission tomography in BD patients indicated the elevation of D2 receptor in several brain regions 60, 61. Of note, different elevation in different region has a subtle effect. Remarkably, one research observed that psychosis was linked with higher D2 receptor density in the caudate nucleus 60, suggesting this local D2 receptor density represented for psychosis rather than mood state. Other studies based on postmortem BD patients revealed that the dopamine D2 receptor was upregulated in the DLPFC 62, and antidopaminergic treatment could increase the D2 receptor levels in animals 63. Specifically, the activation of D2 receptors in the PFC was found to be connected to the activity and function of the hippocampus 64, while blockade of D2 receptors in the PFC led to dysfunction of the amygdala 65. Taken together, these findings suggest that the dysregulation of dopamine and its receptors in the frontal-limbic system might be critical in BD pathogenesis. Hence, the shift release of dopamine or its available receptors in the functional regions might provide a reasonable explanation for BD pathophysiology.

Although the exact mechanism in BD remains enigmatic, based on anatomy and its functional evidence, it can be speculated that dopamine and its related receptors might be responsible for the mood instability in BD through the interaction of the amygdala, hippocampus, and PFC. Among all these brain regions, the PFC likely serves as an upstream regulator. Primarily, it has been shown that dopaminergic projections into PFC have an indispensable role in reward processing 66, and its malfunction possibly explains the dysregulated “reward circuit”. Optogenetic functional magnetic resonance imaging research has identified that stimulating dopamine neurons elicited the striatal activity, whereas locally enhanced medial PFC excitability hampered this striatal response and inhibited the behavioral drive for dopaminergic stimulation in rats. This chronic overactivity of the medial PFC further exerted a sustained suppression on natural reward-motivated behaviors, establishing an enduring state of anhedonia 67. Of note, peculiar prefrontal-striatal activity is profoundly observed in the neuroimage evidence of BD, which might be explained by this mentioned optogenetic results. Specifically, dopamine has been shown to modulate the projection from the PFC into the amygdala. Photostimulation of medial PFC D1 terminals in the basolateral amygdala recapitulated the antidepressant effects in forced swim test and novelty suppressed feeding 68. It has been demonstrated that the PFC can regulate the release of dopamine in the mesolimbic system 69-71, which in turn modulates the activity of the NAc that regulates emotion 72. In particular, the PFC indirectly interacts with the amygdala by acting on dopamine neurons in other brain regions, including the ventral tegmental area (VTA) 73 and basal forebrain 74. Of note, depending on dopamine neurons in the VTA, the PFC could also mediate the function of the hippocampus 75-77.

Generally, it was observed that the static aberrant activity and neurotransmitters/receptors are involved in the development of BD, but the dynamic mood switch was rarely explained. Therefore, more investigations are required to identify the cyclical changes in brain activity or neurotransmitter receptors. Considering the translation into neuroscience research, it is feasible to adopt the optogenetic tools to reproduce or reverse emotional abnormalities through manipulation of mentioned brain regions (e.g., PFC and amygdala) or neural transmitter (e.g., dopamine).

## Dysfunction of the SCN and disrupted circadian rhythms

Abnormal circadian rhythms are vital manifestations of BD 78. However, the causal linkage between biological rhythms and emotions has yet to be clarified 79. For quinpirole-induced animal models of BD, one study clarified that individuals with decreased ability to recover from the disrupted circadian rhythm were more susceptible to BD 80. Of note, disrupted circadian rhythms can also induce manic-like behaviors in mice 80, 81. Moreover, lithium, a mood stabilizer widely prescribed in BD therapeutics, can stabilize and normalize the circadian rhythms and has been verified to be an effective therapy for ameliorating and preventing relapse into manic and depressive states of BD 82, which strongly suggests a bidirectional relationship between BD development and the disordered biological rhythm. In particular, a recent study demonstrated that central clock in the brain largely regulated peripheral metabolic rhythms 83. Meanwhile, peripheral metabolism such as gut microbiota, was also found to interact with brain via epigenetic and genetic regulation 84, which deserves more attention in future research.

Considerable evidence has shown that circadian rhythms might be involved in the dysfunction of a region in the anterior hypothalamus named the SCN, which orchestrates daily behavioral and physiological oscillations 85-87. The master circadian pacemaker in the SCN is largely sustained through the activity of cellular- and tissue-level “clocks” and stimuli from the surrounding environment 86, 88-90. Specifically, the functions of central and peripheral biological clocks are mainly realized via regulatory genes located in SCN. These regulatory genes are synchronized by light signals and generate the body endogenous at a 24 hours rhythm 91. Among various circadian pacemaker of the SCN, rhythmic *CLOCK* expression attracts the most attention. The oscillator *CLOCK* gene thus regulate circadian rhythms through a molecular feedback system including both their own transcription and other clock-controlled genes 86 92 93 without strong external influences 94. Intriguingly, it appears that *CLOCK* gene expression is involved in mood regulation in response to SCN signals and environmental stimuli. However, whether this role relies on the SCN remains to be explored 16. Studies have revealed that the dysregulation of *CLOCK* genes may promote the susceptibility to developing BD and influence circadian phenotypes, which accounts for relapse into episodes 95, indicating the causative role of the *CLOCK* gene. Furthermore, a clinical study proposed that circadian rhythms in the circadian clock gene expression and the cortisol alterations of the buccal cell might be utilized for predicting mania and depression episodes in BD patients 96.

Though direct evidence remains scarce, BD patients displayed enhanced functional connections in the SCN-paraventricular nucleus-dorsomedial hypothalamus nucleus 82, suggesting that SCN dysfunction might disrupts the circadian rhythms in BD patients, and drives mood alterations through downstream interactions. In addition, components of the molecular biological clock are also found in extra-SCN regions, such as the hippocampus, amygdala, lateral habenula, NAc, and PFC 97-99, which should be taken into consideration when focusing on SCN clock.

**Figure 2:**
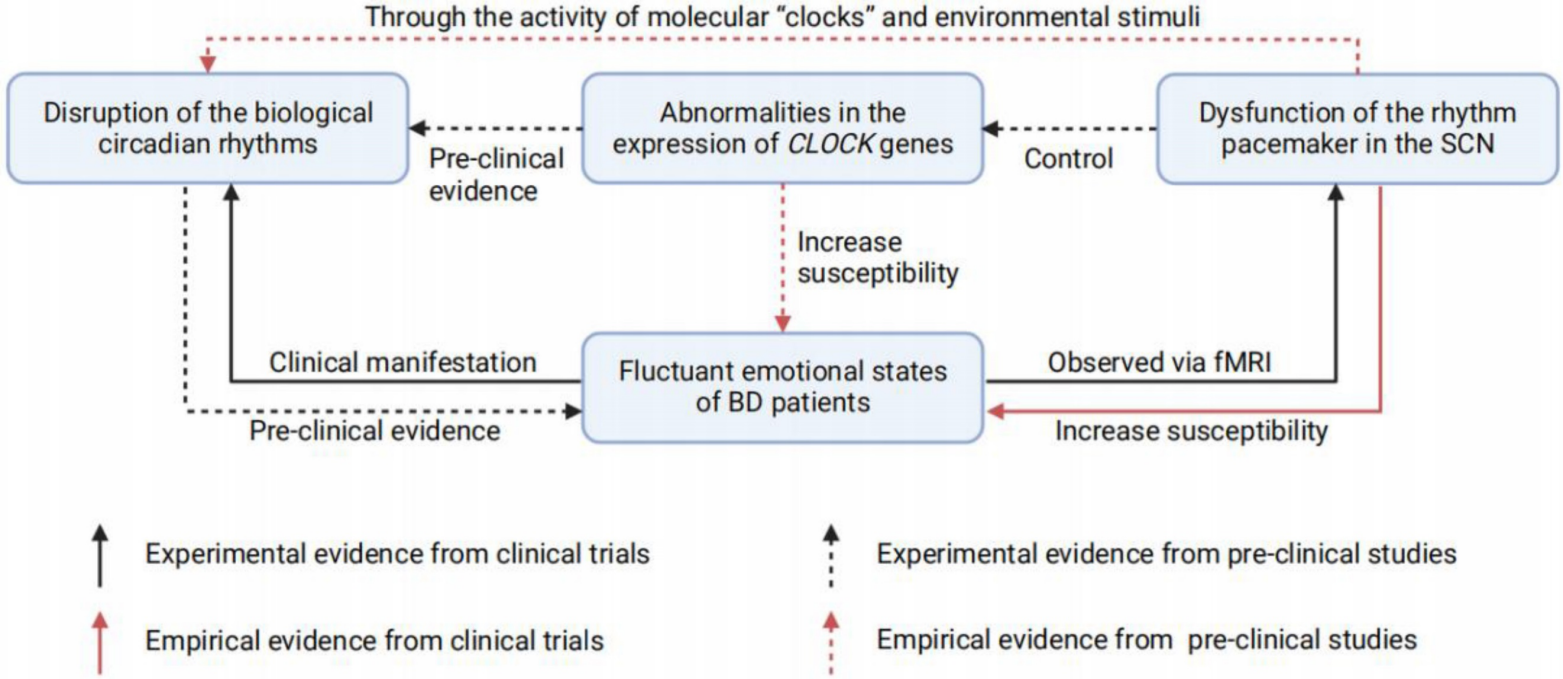
** The relationship between fluctuant emotions of BD, SCN dysfunction, *CLOCK* genes abnormities, and disrupted circadian rhythms.** Disruption of the biological circadian rhythms is commonly found in BD patients, for its correlation with the emotional fluctuation. Pre-clinical studies also demonstrated the disrupted circadian rhythms could lead to depression- or mania-like behaviors in animals. Studies based on fMRI suggest that BD patients have dysfunctions in the SCN rhythm pacemaker, while observed SCN dysfunctions could increase the susceptibility of BD in health population. In addition, pre-clinical studies have also found that there are two ways for the dysfunctional SCN to cause circadian rhythms disruption: i) Through the activity of molecular "clocks" and environmental stimuli; ii) Through the expression of *CLOCK* genes. Of note, abnormalities in the *CLOCK* genes expression could also increase the susceptibility of BD, which has previously been proven pre-clinically. BD = bipolar disorder; SCN = suprachiasmatic nucleus; fMRI = functional magnetic resonance imaging.

In summary, abnormities in the SCN and disrupted biological rhythms are probably involved in BD pathophysiology and thus may act as traits or state markers for BD emotional episodes. (**Figure 2**) In fact, resynchronization and normalization of circadian rhythms has proven effective in BD therapies 100, 101. These findings provide inspiring evidence indicating disrupted rhythm as a contributor to BD, however, they further challenge us to determine which brain regions regulate mood through photic and nonphotic clock alterations. A rhythmic or unrhythmical neural manipulation depending on the light pattern is required to clarify this problem.

## Investigations of optogenetics in BD research

By means of its strong operability, optogenetics allows us to probe a defined brain region or neural circuit disorder in a spatiotemporal manner, thus straddling the field of psychiatry. However, in neuroscience, circuit mechanism discovery depends on the animal model that can be harnessed to utilize the invasive approaches. BD presents complex disorder behaviors including nuanced swing emotional states and disrupted circadian rhythm, making the construction of an animal model more challenging and obstructing the application of optogenetics. Although progression has been made, to date, no animal model has completely mimicked the featured manifestations of BD, including emotional fluctuation and dysregulated biological rhythms 102. Artificially handled animals (stress/drug-induced or gene knocked-out) are often able to simulate only unipolar mania or depression 103, 104, impeding their reliability for the exploration of BD mechanisms. Nevertheless, it is still inspiring that neuroscience techniques are pushing the frontiers of psychiatry. Here, we mainly focus on the aberrant emotion and disrupted circadian rhythm that correlated to the frontal-limbic system and SCN, and suggest the investigations of optogenetics to facilitate the advance of BD research.

One of the known animal models of BD is established based on sleep deprivation, which perturbs the circadian rhythms and models mania episodes in rodents, as elucidated by McClung. Given that sleep disruption stands as a hallmark feature of mania 105, and sleep deprivation has the capacity to incite manic episodes in individuals with bipolar disorder 106, this model shows significance of the validity in combining mood and circadian rhythm. Furthermore, sleep deprivation recapitulated various manic-like behaviors, encompassing insomnia, hyperlocomotion, aggressive interactions with conspecifics, increased sexual mounting behavior, and stereotyped behaviors such as sniffing and rearing 107. Considering the SCN governs a sleep homeostatic process 108, it is undoubtedly an attainable behavioral model to examine their causal link.

Among all existing BD models, it is notably that the *CLOCK*Δ19 mice display a cycling mood profile of mania in the day and euthymia at night, and the elevation of mood coincides with increased excitation of VTA dopaminergic neurons 109. When using RNA interference to specific knock down the *CLOCK* in VTA, mice exhibited hyperactivity and decreased anxiety behavior resembling the *CLOCK*Δ19 mice. However, it also displayed depression behavior in the forced swim and learned helplessness test, leading a mixed manic emotional state, and altered circadian rhythms 110, indicating the interaction between the emotion and the biological circadian rhythms. However, only one optogenetic experiment has been reported. Sidor et al. injected a double-floxed inverted open-reading-frame virus carrying the specific stable step-function opsin in the VTA of TH-Cre transgenic mice to allow direct persistent activation of dopaminergic VTA neurons. After 7 day of optical activation for 1 h per day, mice exhibited mania-like behaviors with less anxiety, which was similar to the behavior of *CLOCK*Δ19 mice 111, suggesting that *CLOCK* may regulate emotion by acting on the activity of dopaminergic VTA neurons. Significantly, this work not only supported dopamine as an important bridge between cycling mood state and rhythm 112, but also established an operatable stimulation paradigm to reproduce the mania phase of BD. Moreover, optogenetic manipulation indicated that the activation of VTA GABAergic neurons (instead of dopaminergic neurons) alleviated dizocilpine induced hyperlocomotion 113, which might be harnessed to reduce the positive psychotic-like behaviors in BD patients. Further investigations should focus on the exact mechanisms that how chronic stimulation of dopaminergic VTA neurons drives the post-manipulation effect, with plasticity and metabolism alterations examined.

One different method to build the BD animal model used electrodes to stimulate the lateral hypothalamus (LHA) with 180 Hz frequency 114. Activating the LHA, rats were successfully induced the mania behavior, including increased sexual self-stimulation, excessive rearing, feeding, and grooming during the kindling interval. Moreover, rats showed hyperlocomotion with decreased rest interval during the mania induction and post-mania days in both light and dark phases. Meanwhile, administration with lithium or valproic acid reversed manic-like behaviors in this model, which further confirmed its validity for BD animal model. Therefore, optogenetic manipulation targeting at the LHA can also be applied to establish a BD animal model. Meanwhile, specific LHA-related circuits can also be dissected to check out which circuit controls the corresponding behavior and how distinct circuits work together in BD development.

Although there is no ideal BD animal model currently, novel intervention measurements with optogenetics can be adopted in advance. In view of the above, we hypothesize a novel investigation for building BD animal model, namely, applying short- or long-term optogenetic stimulation on the rhythm-related brain regions such as the SCN, and then observe the correlated behaviors.

Inspired by the innovative work of the McClung team, herein, we suggest the SCN as a potential optogenetic intervening target, to mimic the phenotype of bipolar disorder and unravel the potential BD mechanisms in modelled animals. Instructive studies applying optogenetics to elucidate the SCN functions are listed in **Table 1**.

On account of the inability of other methods, such as chemical genetics, to simultaneously control spike frequency rhythms, it is important to manipulate the SCN by means of optogenetics in naive mice to simulate dysregulated biological rhythms and examine both timely and post manipulation mood states. Inspiringly, Jones et al. presented protocols for using both *in vitro* and *in vivo* optogenetics to regulate the activity of clock neurons in the SCN to investigate circadian physiology and behavior 115, and these protocols may act as a guide for the investigation of SCN in understanding the mechanism of BD in the future. A study used optogenetic stimulation of vasoactive intestinal peptidergic SCN neurons 116, which is required to transmit light information, *in vivo*. After that, PERIOD 2::LUCIFERASE (PER2::LUC) bioluminescent imaging was carried out to test the functional SCN network *ex vivo*. Long optogenetic activation reproduced the effect of a prolonged photic period in the subsequent longer SCN entrained phase and reduced the SCN free-running period *ex vivo*, and increased the analogous locomotion *in vivo* 117. In addition, virally knocking down another circadian gene *Bmal1* in the SCN led to biological rhythms disruption as well as provoked learned helplessness, behavioral despair, and anxiety phenotypes in mice, which may be independent of light, hinting at the potential role of the SCN in indirect mood regulation 17, 83, 118, 119. Of note, one optogenetic manipulation in the SCN influenced the anxiety phenotype in mice, which reminded us of the importance of the specific activating pattern applied 120.

Intriguingly, there is also one study using optogenetic approaches to enhance the understanding of abnormal attention and impulsion that might happen in BD related to the frontal-limbic system. It focused on the NAc, medial PFC, and their dopaminergic innervation from the VTA 121. This study conducted the 5-choice serial reaction time task and illustrated that the optical activation of the somas in VTA dopaminergic neurons that project to the NAc shell or medial PFC impaired attentive behavior immediately before the presence of cues. However, only the activation of the somas in VTA dopaminergic neurons that project to the NAc core provoked impulsive control behavior 122, hinting BD patients may have deficit in attention and impulse control 123. Therefore, *in vivo* recording such as fiber photometry or electrophysiology within thse projecting specific neural ensembles in BD model is needed to detect the function deficit at circuit level.

Notably, light can modulate behaviors both in rodents and humans, which is particularly evident in reward process. For example, greater reinforcing properties of cocaine are detected during the light than dark-phase of the light/dark cycle in rodents 124, which might be explained by the diurnal changes in dopaminergic activity within the frontal-limbic system 97. Therefore, the intervention of optogenetics should not only consider how neural circuit activity impacts behavior but also how the timing of stimulation influences a given behavioral state 125. In other words, given the overall network synchrony, it should be considered that which rhythm state that optogenetic stimulation is involved, and whether this artificial manipulation is in accordance with the natural biological rhythm of the animals.

Given that fluctuating mood states and disrupted circadian rhythms constitute two hallmark features of BD, it is on an urgent demand to create an ideal animal model, which presents both of these phenotypes. Future studies should focus on modeling more clinical symptoms of BD, which will hopefully provide an accessible approach to explore the neural mechanism. In addition, further investigation on existing animal model is also warranted. To parse the function of the complicated neural basis relevant to BD, proof-of-concept studies in virtue of optogenetics illuminating circuitry mechanisms in detail, thus helping to find potential targets to intervene through precise physical treatment such as deep brain stimulation and repeated transcranial magnetic stimulation.

## Future Directions

### Distinguish the neural substates of BD from other psychiatric morbidities

Challenges remain in BD diagnosis since it is demanding to distinguish the bipolar depression from the unipolar depression or major depressive disorder (MDD). An encouraging finding is that some key reward circuit abnormalities remain consistent in different states of BD, which distinguish it from MDD. A study found that compared with MDD patients or healthy controls, BD patients in the depressive state showed elevated activation in the left VLPFC than MDD patients 126, which had been associated with increased arousal during the processing of the salient emotional stimuli 127. For neurotransmitters, notably, glutamate levels in the frontal lobe were found to be elevated in BD patients but decreased in MDD patients 128, 129. The glutamate pathways in the medial PFC are regulated by dopamine in nonhuman animals 130. Therefore, these abnormities in glutamate levels may also indirectly reflect the dysregulation of dopamine and reward processing. In particular, different levels of glutamate were observed in the distinct mood states of BD patients 131-133, which indicates a potential way to differentiate BD from MDD. Such findings also remain explorations at circuit level in BD condition.

Abnormalities in reward processing have been profoundly involved in BD 134, as well as other mental disorders, including MDD, schizophrenia and autism spectrum disorder 135-137, indicating that these morbidities possibly have structural or functional alteration in shared neural substrate. Despite the overlapping aberrant alternations in neural circuits, there are subtle differences among the segregated reward-related circuits. Fortunately, the application of optogenetic methods, which enable researchers to distinguish differences among reward processing neural circuits under different psychotic states, accelerates progress. For instance, seeking reward persistently regardless of the negative outcomes it brings is a character of mania, while depression is always anhedonia. While the anhedonia behavior has been well excavated, the strengthened reward seeking and attenuated punishment avoiding that can be rescued by BD treatment are far less explored. One study took advantage of optogenetic manipulation revealing that the rostro-medial tegmental nucleus regulated resistance to punishment 138. However, whether this brain region participates in psychotic behavior of BD still remains to explore.

### Investigating the inherent neural abnormalities in different state of BD

The obstacle of BD research lies in the complexity that patients usually show more than two mood states (mania/hypomania, depression, and euthymia) and each state might have different neural substrates. Despite the limitations of the existing animal models, it is feasible to primarily dissect the dysfunctional circuits under mania or depression-like states. Due to the precise control of optogenetics, stimulation with low/moderate/high frequency can induce long-term depression/potentiation in specific synapses. As the latest studies have revealed that the altered synapse plasticity of the lateral habenula is implicated in the dynamic process of getting into depression 139, 140, it is worth considering which cohesive brain regions are rhythmically activated or inhibited to develop into persistent changes in plasticity and thus direct the corresponding behaviors. For instance, the synapse plasticity of SCN-paraventricular nucleus/dorsomedial hypothalamus nucleus can be examined after circadian disruption in some animal models, such as quinpirole-induced mania mice, to determine whether this connection encodes vulnerability to BD 80. In spite of the obscure neural mechanism, the exciting/inhibiting balance 109, 141, the shift in dopamine release 69-71 and its functional receptor 62, 63 and the disrupted biological rhythms 16, 78 and other perspectives based on humans can all be tested in animal models to decipher the mystery of switching between states. Given the shortcomings of symptom-based diagnostics for mental disorders, optogenetic research will doubtlessly be one of the focuses in the future to design treatments tailored for its pathogenesis.

## Summary

To parse the complex neural pathophysiology of BD, proof-of-concept optogenetic explorations are expected to illuminate the obscure pathophysiology at the circuitry level in detail. Focusing on the fluctuating emotion and disrupted rhythms, we gathered previous evidence of the neural activity and neurotransmitter aspects within the frontal-limbic system and the SCN from clinal practice and animals. Then, based on this information, we suggested feasible optogenetic approaches to establish a valid animal model and offer further direction in the dissection of BD pathophysiology. Despite the scarcity of existing research, we provided novel views of the application of optogenetics in BD research, hoping to pave the way for preclinical treatment and identify potential targets for medical intervention.

## Figures and Tables

**Table 1:** Studies using optogenetic methods to reveal the biological functions of SCN.

Time	Animals	Special treatments*	Other research technologies**	Results or conclusions	Ref.
2015	Male and female Drd1a-ChR2 × PER2::LUC mice	-	Immunohistochemistryprotein culture and imaginglocomotor activity monitoring	Optical manipulation of SCN firing rate resets the circadian rhythms *ex vivo* and *in vivo*	142
2016	Male and female Evans ratsMale and female C57BL/6J, VP-Cre knock-in, ChETA, ArchT, and V1aR*^-/-^* mice	Water restriction	Evans blue injectionRetrograde fluorescent microspheresImmunofluorescenceSingle cell RT-PCR	Optogenetic inhibition SCN neurons to decrease the VP release suppresses the firing of OVLT neurons and prevents the corresponding increase in water intake during the anticipatory period.	143
2017	Male and female Opn4*^+/tau‐lacZ^* miceMale and female glutamate decarboxylase 2‐IRES‐Cre mice	Pharmacological manipulation: Ionotropic glutamate receptor blockersVisual stimuli	Immunofluorescence	Optogenetic activation of GHT neurons selectively inhibits the responses to retinal input of SCN and exhibits a day/night difference in a GABAergic manner	144
	Male and female Grpr*^F/y^* miceMale and female Grprw*^t/y^* mice	Selective neurons ablationChemogenetic neurons silencing	ImmunofluorescenceImmunohistochemistry	Optogenetic activation of GRP or GRPR neurons located in the SCN promotes scratching behavior	145
2018	Male and female C57BL/6J miceMale and female VIP-ires-Cre::Rosa-GCaMP5 mice	-	Immunohistochemistry	Optogenetic activation of VIP neuron in SCN with high frequency shifts gene expression rhythms *in vitro* and entrained circadian locomotor rhythms *in vivo*	146
	Male and female C57BL/6J miceMale and female VIP-ires-Cre::Rosa-GCaMP5 mice		Wheel-running test	Glutamatergic and GABA input synapses in ventral SCN neurons exhibit light-dependent plasticity, which can regulate the responsiveness of SCN neurons to the wide range of light input.	147
2019	Male and female homozygous transgenic rats with an arginine vasopressin enhanced green fluorescent protein fusion gene expressed	Retina dissection	Immunohistochemistry	Optogenetic activation of VP-retinal ganglion cells axons projecting to SCN affects the activity of SCN cells through vasopressin.	148
2020	Male and female C57BL/6J miceMale and female AVP-Cre knock-in miceMale and female GAD-Cre mice	NanoTag recording implantation	Immunofluorescence	Optogenetic activation of the GAD-expressing OVLT neurons projecting to VP-expressing SCN neurons mimics the effect that hypertonic saline phase-advances the circadian locomotion	149
	Male and female VIP-CRE, AVP-CRE, GRP-CRE, Syt10-CRE, CKIetau, Bmal1*^fl/fl^*, and cFOS::GFP mice	-	ImmunofluorescenceImmunocytochemistry	Optogenetic control shows that VIP+ SCN neurons increases nighttime instead of daytime sleep	150
	Male and female VIP-IRES-Cre mice		Wheel-running testSleep-wake monitoringImmunohistochemistrySingle-nuclei RNA-sequencing	Optogenetic manipulation clarifies VIP-expressing SCN neurons is required for the locomotor circadian rhythm	151
	Male and female VIP-IRES-Cre miceMale and female ChR2-EYFP miceMale and female Archaerhodopsin 3-EYFP mice	Pharmacological manipulation: VIP receptor antagonist [D-p-Cl-Phe6,Leu17]-VIP, (+)-bicuculline, and ionotropic glutamate receptor blockers	-	Optogenetic manipulation helps reveal that VIP-expressing SCN neurons provided coordinated daily waves through GABAergic input to paraventricular hypothalamus and ventral thalamus, suppressing their activity during the mid to late day	116
	Male and female CRF-Cre, GAD67-Cre, VGAT-IRES-Cre, Rosa26-LSL-tdTomato, and orexin-flp mice	Chemogenetic inhibition of neurons	Sleep and wake recordingsLocomotor activity measurementCalcium imagingImmunofluorescence	Optogenetic activation of the corticotropin-releasing factor neurons in the hypothalamic paraventricular nucleus, one of the output regions of SCN, promotes wakefulness relying orexin neurons in the LHA	152
2021	Male and female Vip::Cre*^+/-^*Per2::Luciferase*^+/-^*, Vip::Cre*^+/-^*floxed-ChR2*^+/-^*, Vip::Cre±Per2::Luciferase*^+/-^*, and Vip::Cre*^+/-^*floxed-ChR2*^+/-^*Per2::Luciferase*^+/-^* miceMale Vip::Cre*^+/-^* mouse	-		Optogenetic activation of VIP-ergic SCN neurons simulating the long photic period induces related locomotor behavior	117
2022	Male heterozygous Vgat-Cre and homozygous Vgat-Cre mice	-	Sleep and wake recordingsBehavioral testsImmunofluorescence	Optogenetic manipulation influences the anxiety-like behavior	153
	Male and female Tg21-31Koba, Dat1-Ires-Cre, Drd1-GFP, and Drd2-GFP mice		ImmunohistochemistryLaser-scanning microscopyElectron microscopyLight-sheet microscopyRNA-scope *in situ* hybridizationCalcium imagingBehavioral tests	Optogenetics helps clarify that SCN send the neuropeptide to dopamine neurons in periventricular nucleus, which innervates lateral septum to control locomotion	120

*The artificial intervention in the experimental group (animals or brain slices) except for gene knock-out (or knock-in), stereotactic injection (virus or other pharmacological intervention) or optogenetic stimulation. **The research technologies used in the study except for optogenetic methods, stereotactic injection (virus or other pharmacological intervention), neuronal electrophysiology, and statistical analysis. SCN = suprachiasmatic nucleus; VIP = vasoactive intestinal peptide; VP = vasopressin; OVLT = organum vasculosum lamina terminalis; PCR = polymerase chain reaction; AVP = arginine-vasopressin (The full name of the gene will not be indicated here).

## Abbreviations

BD: bipolar disorder
MDD: major depressive disorder
SCN: suprachiasmatic nucleus
PFC: prefrontal cortex
DLPFC: dorsolateral prefrontal cortex
VLPFC: ventrolateral prefrontal cortex
ACC: anterior cingulate cortex
NAc: nucleus accumbens
VTA: ventral tegmental area
LHA: lateral hypothalamic area
OVLT: organum vasculosum laminae terminalis
GABA: γ-aminobutyric acid
PER2::LUC: PERIOD 2::LUCIFERASE
VIP: vasoactive intestinal peptide
VP: vasopressin
PCR: polymerase chain reaction
AVP: arginine-vasopressin

## References

[1] Ching CRK, Hibar DP, Gurholt TP, Nunes A, Thomopoulos SI, Abe C (2022). What we learn about bipolar disorder from large-scale neuroimaging: Findings and future directions from the ENIGMA Bipolar Disorder Working Group. Hum Brain Mapp.

[2] Tursini K, Le Cam S, Schwan R, Gross G, Angioi-Duprez K, Conart JB (2022). Visual electrophysiology and neuropsychology in bipolar disorders: A review on current state and perspectives. Neurosci Biobehav Rev.

[3] Anderson IM, Haddad PM, Scott J (2012). Bipolar disorder. BMJ.

[4] Fornaro M, Anastasia A, Monaco F, Novello S, Fusco A, Iasevoli F (2018). Clinical and psychopathological features associated with treatment-emergent mania in bipolar-II depressed outpatients exposed to antidepressants. J Affect Disord.

[5] De Berardis D, Vellante F, Pettorruso M, Lucidi L, Tambelli A, Di Muzio I (2021). Suicide and Genetic Biomarkers: Toward Personalized Tailored-treatment with Lithium and Clozapine. Curr Pharm Des.

[6] Hirschfeld RM, Lewis L, Vornik LA (2003). Perceptions and impact of bipolar disorder: how far have we really come? Results of the national depressive and manic-depressive association 2000 survey of individuals with bipolar disorder. J Clin Psychiatry.

[7] Kato T, Kubota M, Kasahara T (2007). Animal models of bipolar disorder. Neurosci Biobehav R.

[8] Shirai F, Hayashi-Takagi A (2017). Optogenetics: Applications in psychiatric research. Psychiatry Clin Neurosci.

[9] Deisseroth K (2011). Optogenetics. Nat Methods.

[10] Gunaydin LA, Yizhar O, Berndt A, Sohal VS, Deisseroth K, Hegemann P (2010). Ultrafast optogenetic control. Nat Neurosci.

[11] Zhang F, Wang LP, Brauner M, Liewald JF, Kay K, Watzke N (2007). Multimodal fast optical interrogation of neural circuitry. Nature.

[12] Duebel J, Marazova K, Sahel JA (2015). Optogenetics. Curr Opin Ophthalmol.

[13] Hausser M (2014). Optogenetics: the age of light. Nat Methods.

[14] Deisseroth K, Feng G, Majewska AK, Miesenbock G, Ting A, Schnitzer MJ (2006). Next-generation optical technologies for illuminating genetically targeted brain circuits. J Neurosci.

[15] Vai B, Bertocchi C, Benedetti F (2019). Cortico-limbic connectivity as a possible biomarker for bipolar disorder: where are we now?. Expert Rev Neurother.

[16] McClung CA (2007). Role for the Clock gene in bipolar disorder. Cold Spring Harb Symp Quant Biol.

[17] McCarthy MJ, Welsh DK (2012). Cellular circadian clocks in mood disorders. J Biol Rhythms.

[18] Sheline YI (2000). 3D MRI studies of neuroanatomic changes in unipolar major depression: the role of stress and medical comorbidity. Biol Psychiatry.

[19] Fuster JM (2001). The prefrontal cortex-an update: time is of the essence. Neuron.

[20] Morris JS, Dolan RJ (2004). Dissociable amygdala and orbitofrontal responses during reversal fear conditioning. Neuroimage.

[21] Kempton MJ, Salvador Z, Munafo MR, Geddes JR, Simmons A, Frangou S (2011). Structural neuroimaging studies in major depressive disorder. Meta-analysis and comparison with bipolar disorder. Arch Gen Psychiatry.

[22] Maletic V, Raison C (2014). Integrated neurobiology of bipolar disorder. Front Psychiatry.

[23] Zhang L, Wu H, Zhang A, Bai T, Ji GJ, Tian Y (2021). Aberrant brain network topology in the frontoparietal-limbic circuit in bipolar disorder: a graph-theory study. Eur Arch Psychiatry Clin Neurosci.

[24] Chen L, Wang Y, Niu C, Zhong S, Hu H, Chen P (2018). Common and distinct abnormal frontal-limbic system structural and functional patterns in patients with major depression and bipolar disorder. Neuroimage Clin.

[25] Baumann B, Bielau H, Krell D, Agelink MW, Diekmann S, Wurthmann C (2002). Circumscribed numerical deficit of dorsal raphe neurons in mood disorders. Psychol Med.

[26] Matthews PR, Harrison PJ (2012). A morphometric, immunohistochemical, and *in situ* hybridization study of the dorsal raphe nucleus in major depression, bipolar disorder, schizophrenia, and suicide. J Affect Disord.

[27] Benes FM, Kwok EW, Vincent SL, Todtenkopf MS (1998). A reduction of nonpyramidal cells in sector CA2 of schizophrenics and manic depressives. Biol Psychiatry.

[28] Konradi C, Zimmerman EI, Yang CK, Lohmann KM, Gresch P, Pantazopoulos H (2011). Hippocampal interneurons in bipolar disorder. Arch Gen Psychiatry.

[29] Frey BN, Andreazza AC, Houenou J, Jamain S, Goldstein BI, Frye MA (2013). Biomarkers in bipolar disorder: A positional paper from the International Society for Bipolar Disorders Biomarkers Task Force. Aust Nz J Psychiat.

[30] Strakowski SM, Adler CM, Almeida J, Altshuler LL, Blumberg HP, Chang KD (2012). The functional neuroanatomy of bipolar disorder: a consensus model. Bipolar Disord.

[31] Shamay-Tsoory SG, Tomer R, Goldsher D, Berger BD, Aharon-Peretz J (2004). Impairment in cognitive and affective empathy in patients with brain lesions: anatomical and cognitive correlates. J Clin Exp Neuropsychol.

[32] Sun N, Li Y, Zhang A, Yang C, Liu P, Liu Z (2020). Fractional amplitude of low-frequency fluctuations and gray matter volume alterations in patients with bipolar depression. Neurosci Lett.

[33] Xu K, Liu H, Li H, Tang Y, Womer F, Jiang X (2014). Amplitude of low-frequency fluctuations in bipolar disorder: a resting state fMRI study. J Affect Disord.

[34] Chen CH, Suckling J, Lennox BR, Ooi C, Bullmore ET (2011). A quantitative meta-analysis of fMRI studies in bipolar disorder. Bipolar Disord.

[35] Sankar A, Purves K, Colic L, Cox Lippard ET, Millard H, Fan S (2021). Altered frontal cortex functioning in emotion regulation and hopelessness in bipolar disorder. Bipolar Disord.

[36] Zilverstand A, Parvaz MA, Goldstein RZ (2017). Neuroimaging cognitive reappraisal in clinical populations to define neural targets for enhancing emotion regulation. A systematic review. Neuroimage.

[37] Murphy ER, Barch DM, Pagliaccio D, Luby JL, Belden AC (2016). Functional connectivity of the amygdala and subgenual cingulate during cognitive reappraisal of emotions in children with MDD history is associated with rumination. Dev Cogn Neurosci.

[38] Fitzgerald JM, Klumpp H, Langenecker S, Phan KL (2019). Transdiagnostic neural correlates of volitional emotion regulation in anxiety and depression. Depress Anxiety.

[39] Joels M, Baram TZ (2009). The neuro-symphony of stress. Nat Rev Neurosci.

[40] Townsend J, Altshuler LL (2012). Emotion processing and regulation in bipolar disorder: a review. Bipolar Disord.

[41] Yurgelun-Todd DA, Gruber SA, Kanayama G, Killgore WD, Baird AA, Young AD (2000). fMRI during affect discrimination in bipolar affective disorder. Bipolar Disord.

[42] Chang K, Garrett A, Kelley R, Howe M, Sanders EM, Acquaye T (2017). Anomalous prefrontal-limbic activation and connectivity in youth at high-risk for bipolar disorder. J Affect Disord.

[43] Altshuler LL, Bartzokis G, Grieder T, Curran J, Mintz J (1998). Amygdala enlargement in bipolar disorder and hippocampal reduction in schizophrenia: an MRI study demonstrating neuroanatomic specificity. Arch Gen Psychiatry.

[44] Strakowski SM, DelBello MP, Sax KW, Zimmerman ME, Shear PK, Hawkins JM (1999). Brain magnetic resonance imaging of structural abnormalities in bipolar disorder. Arch Gen Psychiatry.

[45] Almeida JR, Versace A, Hassel S, Kupfer DJ, Phillips ML (2010). Elevated amygdala activity to sad facial expressions: a state marker of bipolar but not unipolar depression. Biol Psychiatry.

[46] Lagopoulos J, Malhi GS (2007). A functional magnetic resonance imaging study of emotional Stroop in euthymic bipolar disorder. Neuroreport.

[47] Chen CH, Suckling J, Ooi C, Jacob R, Lupson V, Bullmore ET (2010). A longitudinal fMRI study of the manic and euthymic states of bipolar disorder. Bipolar Disord.

[48] Surguladze SA, Marshall N, Schulze K, Hall MH, Walshe M, Bramon E (2010). Exaggerated neural response to emotional faces in patients with bipolar disorder and their first-degree relatives. Neuroimage.

[49] Mullin BC, Perlman SB, Versace A, de Almeida JR, Labarbara EJ, Klein C (2012). An fMRI study of attentional control in the context of emotional distracters in euthymic adults with bipolar disorder. Psychiatry Res.

[50] Comte M, Schon D, Coull JT, Reynaud E, Khalfa S, Belzeaux R (2016). Dissociating Bottom-Up and Top-Down Mechanisms in the Cortico-Limbic System during Emotion Processing. Cereb Cortex.

[51] Foland LC, Altshuler LL, Bookheimer SY, Eisenberger N, Townsend J, Thompson PM (2008). Evidence for deficient modulation of amygdala response by prefrontal cortex in bipolar mania. Psychiatry Res.

[52] Cerullo MA, Fleck DE, Eliassen JC, Smith MS, DelBello MP, Adler CM (2012). A longitudinal functional connectivity analysis of the amygdala in bipolar I disorder across mood states. Bipolar Disord.

[53] Ong WY, Stohler CS, Herr DR (2019). Role of the Prefrontal Cortex in Pain Processing. Mol Neurobiol.

[54] Ashok AH, Marques TR, Jauhar S, Nour MM, Goodwin GM, Young AH (2017). The dopamine hypothesis of bipolar affective disorder: the state of the art and implications for treatment. Mol Psychiatry.

[55] Nusslock R, Young CB, Damme KS (2014). Elevated reward-related neural activation as a unique biological marker of bipolar disorder: assessment and treatment implications. Behav Res Ther.

[56] Linke J, King AV, Rietschel M, Strohmaier J, Hennerici M, Gass A (2012). Increased medial orbitofrontal and amygdala activation: evidence for a systems-level endophenotype of bipolar I disorder. Am J Psychiatry.

[57] Redlich R, Dohm K, Grotegerd D, Opel N, Zwitserlood P, Heindel W (2015). Reward Processing in Unipolar and Bipolar Depression: A Functional MRI Study. Neuropsychopharmacology.

[58] Bootsman F, Brouwer RM, Schnack HG, van Baal GC, van der Schot AC, Vonk R (2015). Genetic and environmental influences on cortical surface area and cortical thickness in bipolar disorder. Psychol Med.

[59] Gonul AS, Coburn K, Kula M (2009). Cerebral blood flow, metabolic, receptor, and transporter changes in bipolar disorder: the role of PET and SPECT studies. Int Rev Psychiatry.

[60] Pearlson GD, Wong DF, Tune LE, Ross CA, Chase GA, Links JM (1995). *In vivo* D2 dopamine receptor density in psychotic and nonpsychotic patients with bipolar disorder. Arch Gen Psychiatry.

[61] Wong DF, Pearlson GD, Tune LE, Young LT, Meltzer CC, Dannals RF (1997). Quantification of neuroreceptors in the living human brain: IV. Effect of aging and elevations of D2-like receptors in schizophrenia and bipolar illness. J Cereb Blood Flow Metab.

[62] Zhan L, Kerr JR, Lafuente MJ, Maclean A, Chibalina MV, Liu B (2011). Altered expression and coregulation of dopamine signalling genes in schizophrenia and bipolar disorder. Neuropathol Appl Neurobiol.

[63] Ginovart N, Wilson AA, Hussey D, Houle S, Kapur S (2009). D2-receptor upregulation is dependent upon temporal course of D2-occupancy: a longitudinal [11C]-raclopride PET study in cats. Neuropsychopharmacology.

[64] Goto Y, Grace AA (2008). Dopamine modulation of hippocampal-prefrontal cortical interaction drives memory-guided behavior. Cereb Cortex.

[65] Quirk GJ, Mueller D (2008). Neural mechanisms of extinction learning and retrieval. Neuropsychopharmacology.

[66] Schultz W (2010). Dopamine signals for reward value and risk: basic and recent data. Behav Brain Funct.

[67] Ferenczi EA, Zalocusky KA, Liston C, Grosenick L, Warden MR, Amatya D (2016). Prefrontal cortical regulation of brainwide circuit dynamics and reward-related behavior. Science.

[68] Hare BD, Shinohara R, Liu RJ, Pothula S, DiLeone RJ, Duman RS (2019). Optogenetic stimulation of medial prefrontal cortex Drd1 neurons produces rapid and long-lasting antidepressant effects. Nat Commun.

[69] Taber MT, Fibiger HC (1995). Electrical stimulation of the prefrontal cortex increases dopamine release in the nucleus accumbens of the rat: modulation by metabotropic glutamate receptors. J Neurosci.

[70] Beyer CE, Steketee JD (1999). Dopamine depletion in the medial prefrontal cortex induces sensitized-like behavioral and neurochemical responses to cocaine. Brain Res.

[71] Del Arco A, Mora F (2005). Glutamate-dopamine *in vivo* interaction in the prefrontal cortex modulates the release of dopamine and acetylcholine in the nucleus accumbens of the awake rat. J Neural Transm (Vienna).

[72] Del Arco A, Mora F (2009). Neurotransmitters and prefrontal cortex-limbic system interactions: implications for plasticity and psychiatric disorders. J Neural Transm (Vienna).

[73] Hasue RH, Shammah-Lagnado SJ (2002). Origin of the dopaminergic innervation of the central extended amygdala and accumbens shell: a combined retrograde tracing and immunohistochemical study in the rat. J Comp Neurol.

[74] Zaborszky L, Gaykema RP, Swanson DJ, Cullinan WE (1997). Cortical input to the basal forebrain. Neuroscience.

[75] Geisler S, Derst C, Veh RW, Zahm DS (2007). Glutamatergic afferents of the ventral tegmental area in the rat. J Neurosci.

[76] Carr DB, Sesack SR (2000). Projections from the rat prefrontal cortex to the ventral tegmental area: target specificity in the synaptic associations with mesoaccumbens and mesocortical neurons. J Neurosci.

[77] Zornoza T, Cano-Cebrian MJ, Miquel M, Aragon C, Polache A, Granero L (2005). Hippocampal dopamine receptors modulate the motor activation and the increase in dopamine levels in the rat nucleus accumbens evoked by chemical stimulation of the ventral hippocampus. Neuropsychopharmacology.

[78] Pinho M, Sehmbi M, Cudney LE, Kauer-Sant'anna M, Magalhaes PV, Reinares M (2016). The association between biological rhythms, depression, and functioning in bipolar disorder: a large multi-center study. Acta Psychiatr Scand.

[79] Melo MCA, Abreu RLC, Linhares Neto VB, de Bruin PFC, de Bruin VMS (2017). Chronotype and circadian rhythm in bipolar disorder: A systematic review. Sleep Med Rev.

[80] Jung SH, Park JM, Moon E, Chung YI, Lee BD, Lee YM (2014). Delay in the recovery of normal sleep-wake cycle after disruption of the light-dark cycle in mice: a bipolar disorder-prone animal model?. Psychiatry Investig.

[81] Benedetti F, Fresi F, Maccioni P, Smeraldi E (2008). Behavioural sensitization to repeated sleep deprivation in a mice model of mania. Behav Brain Res.

[82] Gold AK, Kinrys G (2019). Treating Circadian Rhythm Disruption in Bipolar Disorder. Curr Psychiatry Rep.

[83] Petrus P, Smith JG, Koronowski KB, Chen S, Sato T, Greco CM (2022). The central clock suffices to drive the majority of circulatory metabolic rhythms. Sci Adv.

[84] Zalar B, Haslberger A, Peterlin B (2018). The Role of Microbiota in Depression - a brief review. Psychiatr Danub.

[85] Boehler NA, Fung SW, Hegazi S, Cheng AH, Cheng HM (2021). Sox2 Ablation in the Suprachiasmatic Nucleus Perturbs Anxiety- and Depressive-like Behaviors. Neurol Int.

[86] Rosenwasser AM, Turek FW (2015). Neurobiology of Circadian Rhythm Regulation. Sleep Med Clin.

[87] Sollars PJ, Pickard GE (2015). The Neurobiology of Circadian Rhythms. Psychiatr Clin North Am.

[88] Wehrens SMT, Christou S, Isherwood C, Middleton B, Gibbs MA, Archer SN (2017). Meal Timing Regulates the Human Circadian System. Curr Biol.

[89] Richards J, Gumz ML (2012). Advances in understanding the peripheral circadian clocks. FASEB J.

[90] Harvey AG (2008). Sleep and circadian rhythms in bipolar disorder: seeking synchrony, harmony, and regulation. Am J Psychiatry.

[91] Jakubowicz D, Wainstein J, Tsameret S, Landau Z (2021). Role of High Energy Breakfast "Big Breakfast Diet" in Clock Gene Regulation of Postprandial Hyperglycemia and Weight Loss in Type 2 Diabetes. Nutrients.

[92] Bering T, Hertz H, Rath MF (2020). Rhythmic Release of Corticosterone Induces Circadian Clock Gene Expression in the Cerebellum. Neuroendocrinology.

[93] Cox KH, Takahashi JS (2019). Circadian clock genes and the transcriptional architecture of the clock mechanism. J Mol Endocrinol.

[94] Piggins HD (2002). Human clock genes. Ann Med.

[95] Bellivier F, Geoffroy PA, Etain B, Scott J (2015). Sleep- and circadian rhythm-associated pathways as therapeutic targets in bipolar disorder. Expert Opin Ther Targets.

[96] Moon JH, Cho CH, Son GH, Geum D, Chung S, Kim H (2016). Advanced Circadian Phase in Mania and Delayed Circadian Phase in Mixed Mania and Depression Returned to Normal after Treatment of Bipolar Disorder. EBioMedicine.

[97] Webb IC, Baltazar RM, Wang X, Pitchers KK, Coolen LM, Lehman MN (2009). Diurnal Variations in Natural and Drug Reward, Mesolimbic Tyrosine Hydroxylase, and Clock Gene Expression in the Male Rat. J Biol Rhythm.

[98] McClung CA, Sidiropoulou K, Vitaterna M, Takahashi JS, White FJ, Cooper DC (2005). Regulation of dopaminergic transmission and cocaine reward by the Clock gene. P Natl Acad Sci USA.

[99] Li JZ, Bunney BG, Meng F, Hagenauer MH, Walsh DM, Vawter MP (2013). Circadian patterns of gene expression in the human brain and disruption in major depressive disorder. P Natl Acad Sci USA.

[100] Henriksen TE, Skrede S, Fasmer OB, Schoeyen H, Leskauskaite I, Bjorke-Bertheussen J (2016). Blue-blocking glasses as additive treatment for mania: a randomized placebo-controlled trial. Bipolar Disord.

[101] Barbini B, Benedetti F, Colombo C, Dotoli D, Bernasconi A, Cigala-Fulgosi M (2005). Dark therapy for mania: a pilot study. Bipolar Disord.

[102] Logan RW, McClung CA (2016). Animal models of bipolar mania: The past, present and future. Neuroscience.

[103] Liu W, Xue X, Xia J, Liu J, Qi Z (2018). Swimming exercise reverses CUMS-induced changes in depression-like behaviors and hippocampal plasticity-related proteins. J Affect Disord.

[104] Coque L, Mukherjee S, Cao JL, Spencer S, Marvin M, Falcon E (2011). Specific role of VTA dopamine neuronal firing rates and morphology in the reversal of anxiety-related, but not depression-related behavior in the ClockDelta19 mouse model of mania. Neuropsychopharmacology.

[105] Mansell W, Pedley R (2008). The ascent into mania: a review of psychological processes associated with the development of manic symptoms. Clin Psychol Rev.

[106] Wehr TA (1992). Improvement of depression and triggering of mania by sleep deprivation. JAMA.

[107] Aline S (2017). de Miranda RA, Antônio L. Teixeira. Chapter 43 - Animal Models of Mania: Essential Tools to Better Understand Bipolar Disorder. Animal Models for the Study of Human Disease (Second Edition).

[108] Deboer T, Vansteensel MJ, Detari L, Meijer JH (2003). Sleep states alter activity of suprachiasmatic nucleus neurons. Nat Neurosci.

[109] McClung CA, Sidiropoulou K, Vitaterna M, Takahashi JS, White FJ, Cooper DC (2005). Regulation of dopaminergic transmission and cocaine reward by the Clock gene. Proc Natl Acad Sci U S A.

[110] Mukherjee S, Coque L, Cao JL, Kumar J, Chakravarty S, Asaithamby A (2010). Knockdown of Clock in the ventral tegmental area through RNA interference results in a mixed state of mania and depression-like behavior. Biol Psychiatry.

[111] Sidor MM, Spencer SM, Dzirasa K, Parekh PK, Tye KM, Warden MR (2015). Daytime spikes in dopaminergic activity drive rapid mood-cycling in mice. Mol Psychiatry.

[112] Berk M, Dodd S, Kauer-Sant'anna M, Malhi GS, Bourin M, Kapczinski F (2007). Dopamine dysregulation syndrome: implications for a dopamine hypothesis of bipolar disorder. Acta Psychiatr Scand Suppl.

[113] Lu C, Feng Y, Li H, Gao Z, Zhu X, Hu J (2022). A preclinical study of deep brain stimulation in the ventral tegmental area for alleviating positive psychotic-like behaviors in mice. Front Hum Neurosci.

[114] Abulseoud OA, Camsari UM, Ruby CL, Mohamed K, Abdel Gawad NM, Kasasbeh A (2014). Lateral hypothalamic kindling induces manic-like behavior in rats: a novel animal model. Int J Bipolar Disord.

[115] Jones JR, Tackenberg MC, McMahon DG (2021). Optogenetic Methods for the Study of Circadian Rhythms. Methods Mol Biol.

[116] Paul S, Hanna L, Harding C, Hayter EA, Walmsley L, Bechtold DA (2020). Output from VIP cells of the mammalian central clock regulates daily physiological rhythms. Nat Commun.

[117] Tackenberg MC, Hughey JJ, McMahon DG (2021). Optogenetic stimulation of VIPergic SCN neurons induces photoperiodic-like changes in the mammalian circadian clock. Eur J Neurosci.

[118] Fernandez DC, Fogerson PM, Lazzerini Ospri L, Thomsen MB, Layne RM, Severin D (2018). Light Affects Mood and Learning through Distinct Retina-Brain Pathways. Cell.

[119] Landgraf D, Long JE, Proulx CD, Barandas R, Malinow R, Welsh DK (2016). Genetic Disruption of Circadian Rhythms in the Suprachiasmatic Nucleus Causes Helplessness, Behavioral Despair, and Anxiety-like Behavior in Mice. Biol Psychiatry.

[120] Korchynska S, Rebernik P, Pende M, Boi L, Alpar A, Tasan R (2022). A hypothalamic dopamine locus for psychostimulant-induced hyperlocomotion in mice. Nat Commun.

[121] Boekhoudt L, Voets ES, Flores-Dourojeanni JP, Luijendijk MC, Vanderschuren LJ, Adan RA (2017). Chemogenetic Activation of Midbrain Dopamine Neurons Affects Attention, but not Impulsivity, in the Five-Choice Serial Reaction Time Task in Rats. Neuropsychopharmacology.

[122] Flores-Dourojeanni JP, van Rijt C, van den Munkhof MH, Boekhoudt L, Luijendijk MCM, Vanderschuren L (2021). Temporally Specific Roles of Ventral Tegmental Area Projections to the Nucleus Accumbens and Prefrontal Cortex in Attention and Impulse Control. J Neurosci.

[123] Moeller FG, Barratt ES, Dougherty DM, Schmitz JM, Swann AC (2001). Psychiatric aspects of impulsivity. Am J Psychiatry.

[124] Abarca C, Albrecht U, Spanagel R (2002). Cocaine sensitization and reward are under the influence of circadian genes and rhythm. P Natl Acad Sci USA.

[125] Sidor MM, McClung CA (2014). Timing matters: using optogenetics to chronically manipulate neural circuitry and rhythms. Frontiers in Behavioral Neuroscience.

[126] Chase HW, Nusslock R, Almeida JR, Forbes EE, LaBarbara EJ, Phillips ML (2013). Dissociable patterns of abnormal frontal cortical activation during anticipation of an uncertain reward or loss in bipolar versus major depression. Bipolar Disord.

[127] Dolcos F, LaBar KS, Cabeza R (2004). Dissociable effects of arousal and valence on prefrontal activity indexing emotional evaluation and subsequent memory: an event-related fMRI study. Neuroimage.

[128] Gigante AD, Bond DJ, Lafer B, Lam RW, Young LT, Yatham LN (2012). Brain glutamate levels measured by magnetic resonance spectroscopy in patients with bipolar disorder: a meta-analysis. Bipolar Disord.

[129] Luykx JJ, Laban KG, van den Heuvel MP, Boks MP, Mandl RC, Kahn RS (2012). Region and state specific glutamate downregulation in major depressive disorder: a meta-analysis of (1)H-MRS findings. Neurosci Biobehav Rev.

[130] Kable JW, Glimcher PW (2009). The neurobiology of decision: consensus and controversy. Neuron.

[131] Ongur D, Jensen JE, Prescot AP, Stork C, Lundy M, Cohen BM (2008). Abnormal glutamatergic neurotransmission and neuronal-glial interactions in acute mania. Biol Psychiatry.

[132] Frye MA, Watzl J, Banakar S, O'Neill J, Mintz J, Davanzo P (2007). Increased anterior cingulate/medial prefrontal cortical glutamate and creatine in bipolar depression. Neuropsychopharmacology.

[133] Bhagwagar Z, Wylezinska M, Jezzard P, Evans J, Ashworth F, Sule A (2007). Reduction in occipital cortex gamma-aminobutyric acid concentrations in medication-free recovered unipolar depressed and bipolar subjects. Biol Psychiatry.

[134] Abler B, Greenhouse I, Ongur D, Walter H, Heckers S (2008). Abnormal reward system activation in mania. Neuropsychopharmacology.

[135] Whitton AE, Treadway MT, Pizzagalli DA (2015). Reward processing dysfunction in major depression, bipolar disorder and schizophrenia. Curr Opin Psychiatry.

[136] Hu H (2016). Reward and Aversion. Annu Rev Neurosci.

[137] Chevallier C, Kohls G, Troiani V, Brodkin ES, Schultz RT (2012). The social motivation theory of autism. Trends Cogn Sci.

[138] Vento PJ, Burnham NW, Rowley CS, Jhou TC (2017). Learning From One's Mistakes: A Dual Role for the Rostromedial Tegmental Nucleus in the Encoding and Expression of Punished Reward Seeking. Biol Psychiatry.

[139] Hu H, Cui Y, Yang Y (2020). Circuits and functions of the lateral habenula in health and in disease. Nat Rev Neurosci.

[140] Cerniauskas I, Winterer J, de Jong JW, Lukacsovich D, Yang H, Khan F (2019). Chronic Stress Induces Activity, Synaptic, and Transcriptional Remodeling of the Lateral Habenula Associated with Deficits in Motivated Behaviors. Neuron.

[141] Li L, Zhang LZ, He ZX, Ma H, Zhang YT, Xun YF (2021). Dorsal raphe nucleus to anterior cingulate cortex 5-HTergic neural circuit modulates consolation and sociability. Elife.

[142] Jones JR, Tackenberg MC, McMahon DG (2015). Manipulating circadian clock neuron firing rate resets molecular circadian rhythms and behavior. Nat Neurosci.

[143] Gizowski C, Zaelzer C, Bourque CW (2016). Clock-driven vasopressin neurotransmission mediates anticipatory thirst prior to sleep. Nature.

[144] Hanna L, Walmsley L, Pienaar A, Howarth M, Brown TM (2017). Geniculohypothalamic GABAergic projections gate suprachiasmatic nucleus responses to retinal input. J Physiol.

[145] Yu YQ, Barry DM, Hao Y, Liu XT, Chen ZF (2017). Molecular and neural basis of contagious itch behavior in mice. Science.

[146] Mazuski C, Abel JH, Chen SP, Hermanstyne TO, Jones JR, Simon T (2018). Entrainment of Circadian Rhythms Depends on Firing Rates and Neuropeptide Release of VIP SCN Neurons. Neuron.

[147] Cheng J, Huang X, Liang Y, Xue T, Wang L, Bao J (2018). Plasticity of Light-induced Concurrent Glutamatergic and GABAergic Quantal Events in the Suprachiasmatic Nucleus. J Biol Rhythms.

[148] Hume C, Allchorne A, Grinevich V, Leng G, Ludwig M (2019). Effects of optogenetic stimulation of vasopressinergic retinal afferents on suprachiasmatic neurones. J Neuroendocrinol.

[149] Gizowski C, Bourque CW (2020). Sodium regulates clock time and output via an excitatory GABAergic pathway. Nature.

[150] Collins B, Pierre-Ferrer S, Muheim C, Lukacsovich D, Cai Y, Spinnler A (2020). Circadian VIPergic Neurons of the Suprachiasmatic Nuclei Sculpt the Sleep-Wake Cycle. Neuron.

[151] Todd WD, Venner A, Anaclet C, Broadhurst RY, De Luca R, Bandaru SS (2020). Suprachiasmatic VIP neurons are required for normal circadian rhythmicity and comprised of molecularly distinct subpopulations. Nat Commun.

[152] Ono D, Mukai Y, Hung CJ, Chowdhury S, Sugiyama T, Yamanaka A (2020). The mammalian circadian pacemaker regulates wakefulness via CRF neurons in the paraventricular nucleus of the hypothalamus. Sci Adv.

[153] Vadnie CA, Petersen KA, Eberhardt LA, Hildebrand MA, Cerwensky AJ, Zhang H (2021). The Suprachiasmatic Nucleus Regulates Anxiety-Like Behavior in Mice. Front Neurosci.

